# Relative fat mass is associated with vitamin D deficiency in individuals with diabetes: evidence from NHANES and a Chinese cohort

**DOI:** 10.3389/fendo.2025.1659361

**Published:** 2025-10-01

**Authors:** Qichao Yang, Mengjiao Xu, Lu Qin, Xuejing Shao, Han Yan

**Affiliations:** Department of Endocrinology, Wujin Clinical College of Xuzhou Medical University, Affiliated Wujin Hospital of Jiangsu University, Changzhou, Jiangsu, China

**Keywords:** relative fat mass, vitamin D deficiency, diabetes, NHANES, bodycomposition

## Abstract

**Purpose:**

Relative fat mass (RFM) is a new metric used for obesity assessment. We aim to investigate the association between RFM and vitamin D deficiency in patients with diabetes.

**Methods:**

A total of 5,128 participants with diabetes mellitus from the NHANES 2007–2018 and an external Chinese validation cohort of 238 subjects from the Affiliated Wujin Hospital of Jiangsu University were analyzed. Logistic and linear regression, subgroup and curve fitting analyses were performed to assess the relationships between RFM and vitamin D deficiency risk as well as serum 25(OH)D levels. Receiver operating characteristic (ROC) and decision curve analysis (DCA) were applied to compare diagnostic efficacy among RFM, body mass index (BMI), waist circumference (WC), and height.

**Results:**

Vitamin D deficiency prevalence increased with rising RFM levels (P<0.001). Higher RFM was significantly associated with increased risk of vitamin D deficiency (OR = 1.056, 95%CI= (1.039, 1.073), P<0.001) and lower 25(OH)D levels (β=-0.662, 95%CI= (-0.852, -0.471), P<0.001) in patients with diabetes. ROC and DCA indicated that RFM yielded the highest discrimination for vitamin D deficiency (AUC = 0.626), outperforming BMI (0.592), WC (0.567), and height (0.492). The associations remained robust in various subgroups and were confirmed in the external Chinese population.

**Conclusions:**

RFM is superior to conventional obesity measures in identifying individuals with diabetes at high risk for vitamin D deficiency. RFM may help to improve clinical risk stratification and management.

## 1 Introduction

Obesity, marked by excessive fat accumulation, has emerged as a leading global health concern in the 21st century (1). This chronic and multifaceted condition greatly increases the risk of non-communicable diseases such as hypertension, cardiovascular disorders, type 2 diabetes, and certain cancers, thereby escalating healthcare costs and diminishing patients’ quality of life (2–4).

Although body mass index (BMI) is frequently used for its ease of calculation, it indirectly assesses body composition and cannot differentiate between lean tissue and adipose tissue or provide information on fat distribution (5). These shortcomings may result in inaccurate assessment of metabolic risk (6). While advanced imaging techniques such as Computed Tomography (CT) and Magnetic Resonance Imaging (MRI) offer greater accuracy, their cost, accessibility, and radiation risks limit their routine clinical application (7, 8). Therefore, there is an ongoing need for more accurate and practical approaches to measure body fat. The Relative fat mass (RFM) index, based on accessible measures of height and waist circumference (WC), has proven to correlate well with body fat percentage as determined by Dual-energy X-ray Absorptiometry (DXA) scans (9, 10). Existing literature also suggests that RFM demonstrates a stronger association with metabolic disease risks-including diabetes, non-alcoholic fatty liver disease (NAFLD), cardiovascular diseases, and depression-than either BMI or WC (11–15).

On the other hand, apart from its established functions in maintaining calcium balance and bone health, vitamin D is now recognized for its significant roles in immune modulation, inflammatory processes, and metabolism (16, 17). Globally, more than one-third of the population is estimated to be vitamin D deficient, likely amplified by modern lifestyles. Numerous observational studies have identified a consistent inverse association between serum 25-hydroxyvitamin D [25(OH)D] concentrations and obesity, typically measured by BMI (18–20). Serum 25(OH)D varies with BMI and absolute body weight (21). In pooled multivariable models, a per−kg−per−day vitamin D dose explained ~34.5% of circulating 25(OH)D variance, yielding clear BMI-related disparities (21). Obese and overweight adults averaged ~20 and ~8 nmol/L lower concentration of 25(OH)D, respectively, necessitating ~2.6 and 1.47 times higher vitamin D supplementation (21). Nonetheless, relying on BMI as the primary index may obscure the complex relationship between true adiposity and vitamin D status. Unlike BMI, which primarily reflects overall body mass, the RFM incorporates waist circumference, height, and sex, and therefore aligns more closely with adipose depots-particularly central fat distribution-that are biologically relevant to vitamin D metabolism. Conceptually, this should enhance its ability to identify individuals at high risk of vitamin D deficiency (10). Thus, the present study seeks to elucidate the association between RFM and vitamin D deficiency in individuals with diabetes mellitus.

## 2 Materials and methods

### 2.1 Participants

This study draws on data from the National Health and Nutrition Examination Survey (NHANES), a reputable and publicly accessible source extensively used in health research worldwide. NHANES protocols were approved by the National Center for Health Statistics Research Ethics Review Board, and informed consent was obtained from participants at the time of the original NHANES data collection (22). Our study is a secondary analysis of de-identified public-use NHANES data. The analysis includes combined data from six NHANES cycles (2007-2018), encompassing 59,842 individuals. Participants were excluded if they were under 20 years old, pregnant, did not have diabetes, or lacked RFM or vitamin D data. Diabetes was identified through self-reported diagnosis, fasting plasma glucose (FPG) ≥7.0 mmol/L, hemoglobin A1c (HbA1c) ≥6.5%, or current antidiabetic medication use. After applying these criteria, 5,128 participants with diabetes mellitus were included in the final analysis.

External validation utilized data from diabetes patients enrolled in health education programs at the Department of Endocrinology, the Affiliated Wujing Hospital of Jiangsu University, from January 2024 to January 2025. Diagnosis was confirmed using to the American Diabetes Association criteria (23). The validation cohort consisted of 238 participants (124 men and 114 women), aged 31–88 years (median age 62). The study received Ethics Committee approval of Affiliated Wujin Hospital of Jiangsu University (Protocol: 2025-SR-086), and all participants provided written consent.

### 2.2 Exposure and outcome

This study considered RFM as the exposure, determined by the formula RFM = 64 − (20 × height/WC) + (12 × sex), where sex is 0 for males and 1 for females (10). The formula was empirically derived using DXA-measured body fat percentage as the criterion measure and validated in independent cohorts (10). Serum 25(OH)D (sum of 25(OH)D2 and 25(OH)D3) was the outcome variable (24). For patients with diabetes from NHANES 2007-2018, 25(OH)D levels were obtained using liquid chromatography-tandem mass spectrometry (LC-MS/MS). For diabetic patients from the Affiliated Wujin Hospital of Jiangsu University, serum 25(OH)D levels were measured using a chemiluminescence assay (Siemens ADVIA Centaur XP, Germany). The criterion for vitamin D deficiency was a serum 25(OH)D concentration under 50 nmol/L (20 ng/mL), according to Endocrine Society guidelines (25).

### 2.3 Covariates

Covariates in the NHANES included demographics (age, sex, race, poverty to income ratio [PIR], education, smoking status, alcohol use), physical metrics (BMI, height, WC), laboratory results (hemoglobin A1c [HbA1c], triglycerides [TG], total cholesterol [TC], high- and low-density lipoprotein cholesterol [HDL-c, LDL-c], serum creatinine [SCr]), and medical history (hypertension, cardiovascular disease [CVD]). Race was grouped as Mexican American, non-Hispanic Black, non-Hispanic White, other Hispanic, and other. Education was classified as <high school, high school, or >high school. Smoking (former/current) and alcohol use (≥12 drinks per year) were recorded. Hypertension was defined by self-report, average systolic blood pressure (SBP) ≥140 mmHg, diastolic blood pressure (DBP) ≥90 mmHg, or antihypertensive use. CVD was based on self-reported heart attack, stroke, heart failure, coronary artery disease, or angina. The cohort of the Affiliated Wujing Hospital of Jiangsu University collected similar data: demographics (age, sex, smoking, alcohol use), medical history (hypertension, CVD), physical measurements (BMI, WC, height), and labs (HbA1c, blood lipids [TC, TG, HDL-c, LDL-c], SCr) from fasting samples.

### 2.5 Statistical analysis

In line with the Centers for Disease Control and Prevention recommendations, NHANES analyses utilized population weights and addressed the complex survey design. Medians with interquartile range described continuous data, while categorical variables were shown as unweighted counts and weighted percentages. Missing data, assumed to be missing at random, were imputed using the random forest method implemented in the R package missForest. The algorithm was run for 10 iterations with forests of 100 trees. Out-of-bag evaluation indicated adequate performance (NRMSE = 0.0003, PFC = 0.0812). Group differences were analyzed using the Kruskal-Wallis test and the chi-square test. Logistic regression provided odds ratios (ORs) and 95% confidence intervals (CIs) for the relationship between RFM and vitamin D deficiency across three models, incrementally adjusting for demographic, socioeconomic, lifestyle, and clinical factors. SHapley Additive exPlanations (SHAP) values were applied to interpret model behavior and quantify the contribution of each feature. Based on the Shapley value concept from game theory, SHAP fairly distributes the contribution of predictors for individual predictions. Visualizations for feature importance and swarm plots were produced. Additionally, linear regression was used to assess the association between RFM and 25(OH) D levels. Smooth curve fitting evaluated possible nonlinear links. Stratification was performed by major effect modifiers, such as age, sex, BMI, hypertension, and CVD. The predictive value of RFM for vitamin D deficiency was tested using receiver operating characteristic (ROC) and decision curve analysis (DCA) curves. A diabetes cohort from the Affiliated Wujing Hospital of Jiangsu University served for external validation and to examine links between RFM and vitamin D deficiency. Analyses were performed in R version 4.2.0; significance set at P<0.05.

## 3 Results

### 3.1 Baseline characteristics

**Supplementary Table 1** presents baseline characteristics of diabetic participants grouped by vitamin D deficiency status. Compared with those without vitamin D deficiency, individuals with deficiency tended to be younger, with a higher proportion of Non-Hispanic Black and Mexican American participants (P<0.001). The vitamin D deficient group also generally had lower PIR and educational attainment (P<0.01). Regarding metabolic risk factors, those with vitamin D deficiency showed higher BMI, WC, HbA1c, TG, TC, LDL-c, and RFM, but lower HDL-c and SCr (P<0.001). Additionally, alcohol use was less common among those with vitamin D deficiency (P<0.01). Baseline characteristics of diabetic participants were analyzed across quartiles of RFM (**Supplementary Table 2**). Higher RFM quartiles were associated with younger age, a higher proportion of females, and significant differences in race, education, PIR, smoking and alcohol use, as well as increased prevalence of hypertension and CVDs (all P < 0.05). Furthermore, anthropometric, and biochemical parameters-including BMI, WC, height, lipid profiles, SCr, and 25(OH)D levels-showed significant variation, with a greater prevalence of vitamin D deficiency observed in participants with higher RFM (all P < 0.01).

### 3.2 RFM and vitamin D deficiency

**Figure 1:**
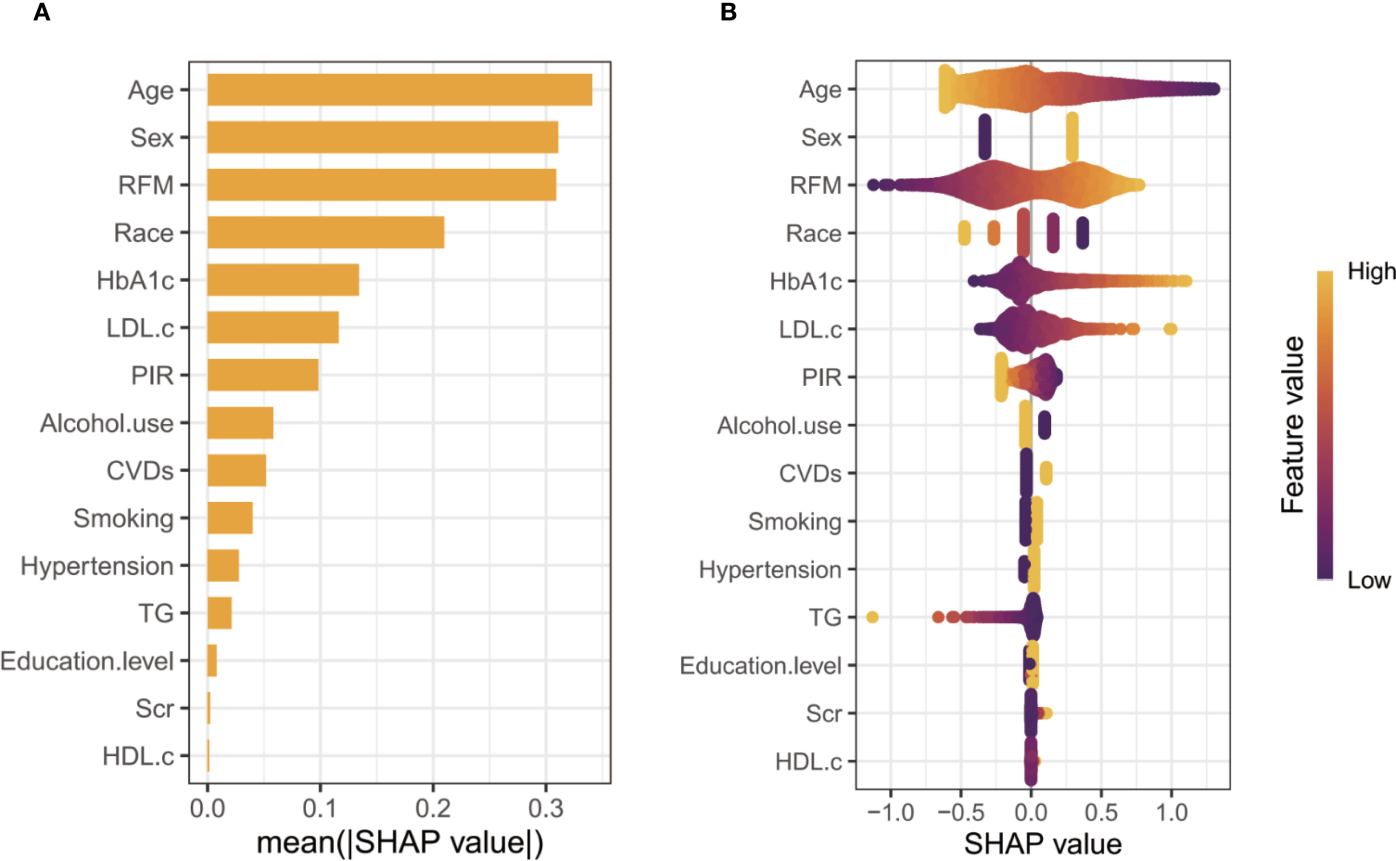
SHAP analysis of feature importance. (**A**, Ranking of features based on mean absolute SHAP values; **B**, Swarm plot illustrating the distribution of SHAP values and their relationship with feature magnitudes).

**Figure 2:**
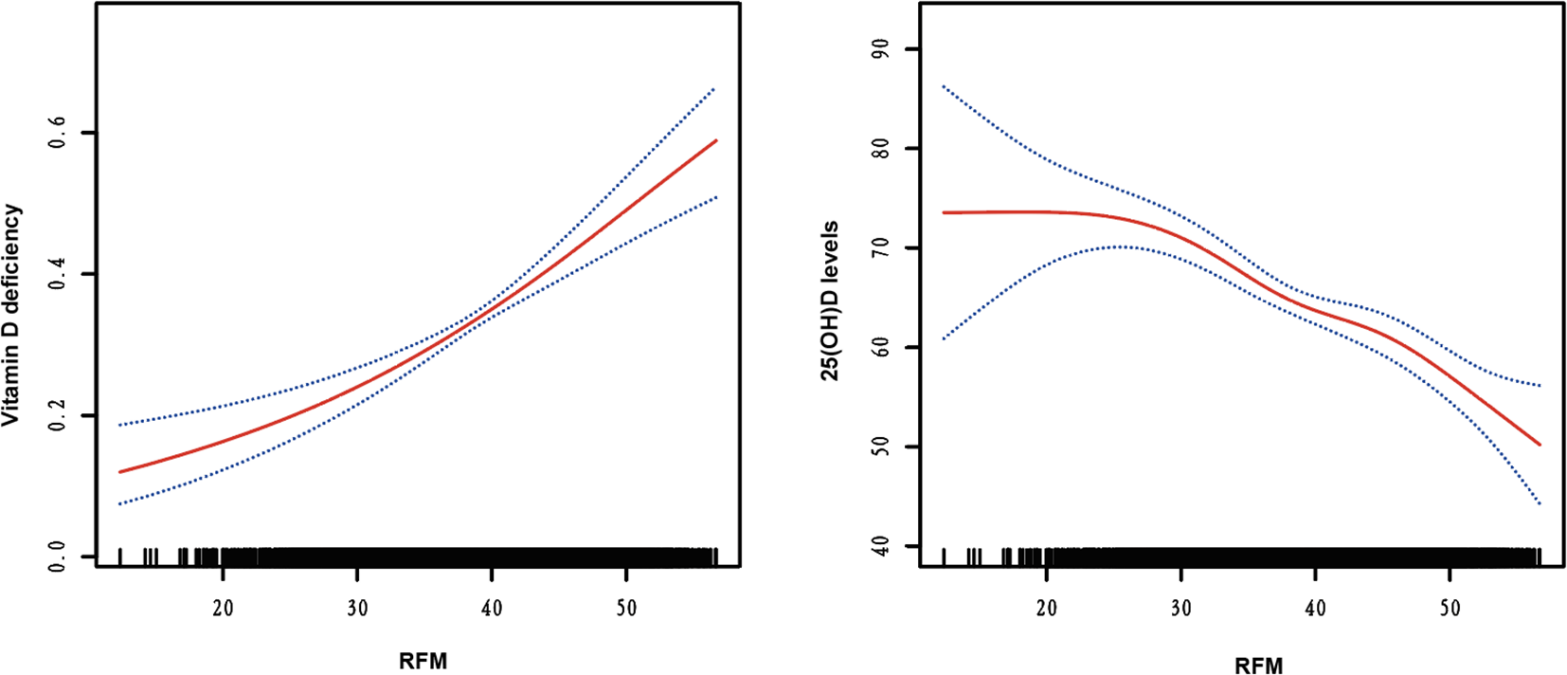
Smooth curve fitting of RFM with risk of vitamin D deficiency and serum 25(OH)D Levels.

**Supplementary Table 3** presents the results of three logistic regression models evaluating the association between RFM levels and vitamin D deficiency risk. Models 1 (non-adjusted) and Model 2 (adjusted for age, sex, race, PIR, education, smoking status, and alcohol use) demonstrate a significant positive trend in vitamin D deficiency with increasing RFM levels (all *P* for < 0.001). In Model 3, after adjusting for potential confounders, including age, sex, race, PIR, education, smoking status, and alcohol use, hypertension, CVDs, HbA1c, TG, LDL-c, HDL-c, and SCr, the ORs and 95% CIs for Q1, Q2, Q3, and Q4 were 1.000, 1.385 (1.153-1.663), 1.707 (1.280-2.278), and 2.642 (1.883-3.708), respectively, with a trend test P-value < 0.001. SHAP analysis identified age as the most influential predictor of vitamin D deficiency, with RFM ranking third in feature importance (**Figure 1A**). The swarm plot demonstrated that higher RFM values (highlighted in yellow) were associated with increased SHAP scores, indicating a greater risk of vitamin D deficiency (**Figure 1B**). In conducting a linear regression analysis with serum 25(OH)D levels as the dependent variable, we similarly discovered a close correlation between RFM and 25(OH)D levels in Model 1 (β= -0.115, 95%CI= (-0.212, -0.017), P<0.022), Model 2 (β=-0.708, 95%CI= (-0.888, -0.528), P<0.001), and Model 3 (β=-0.662, 95%CI= (-0.852, -0.471), P<0.001) (**Supplementary Table 4**). Smooth curve fitting analysis revealed a linear trend between RFM and the risk of vitamin D deficiency and 25(OH)D levels, with no turning point found (**Figure 2**).

### 3.3 Stratified analyses

**Figure 3:**
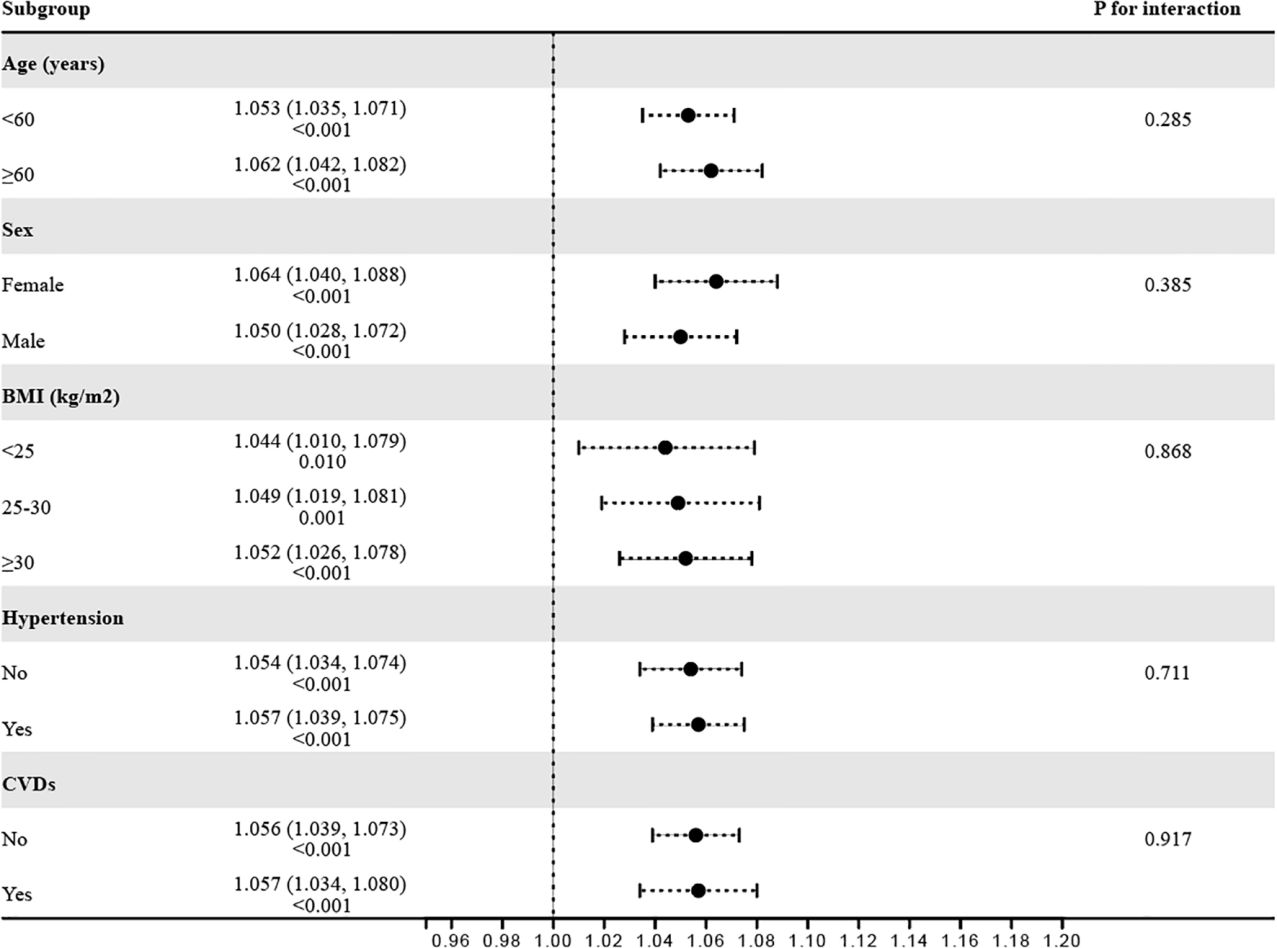
Consistency of the relationship between RFM and vitamin D deficiency across subgroups.

To verify the robustness of the observed correlation between RFM and Vitamin D deficiency, subgroup analyses were conducted based on various stratification variables (**Figure 3**). The results indicated consistent associations across subgroups, including age (<60 years/≥60 years), sex (female/male), BMI (<25 kg/m2/25–30 kg/m2/≥30 kg/m2), hypertension (No/Yes), and CVD (No/Yes), with all P for interaction values exceeding 0.05.

### 3.4 Clinical utility of RFM

**Figure 4:**
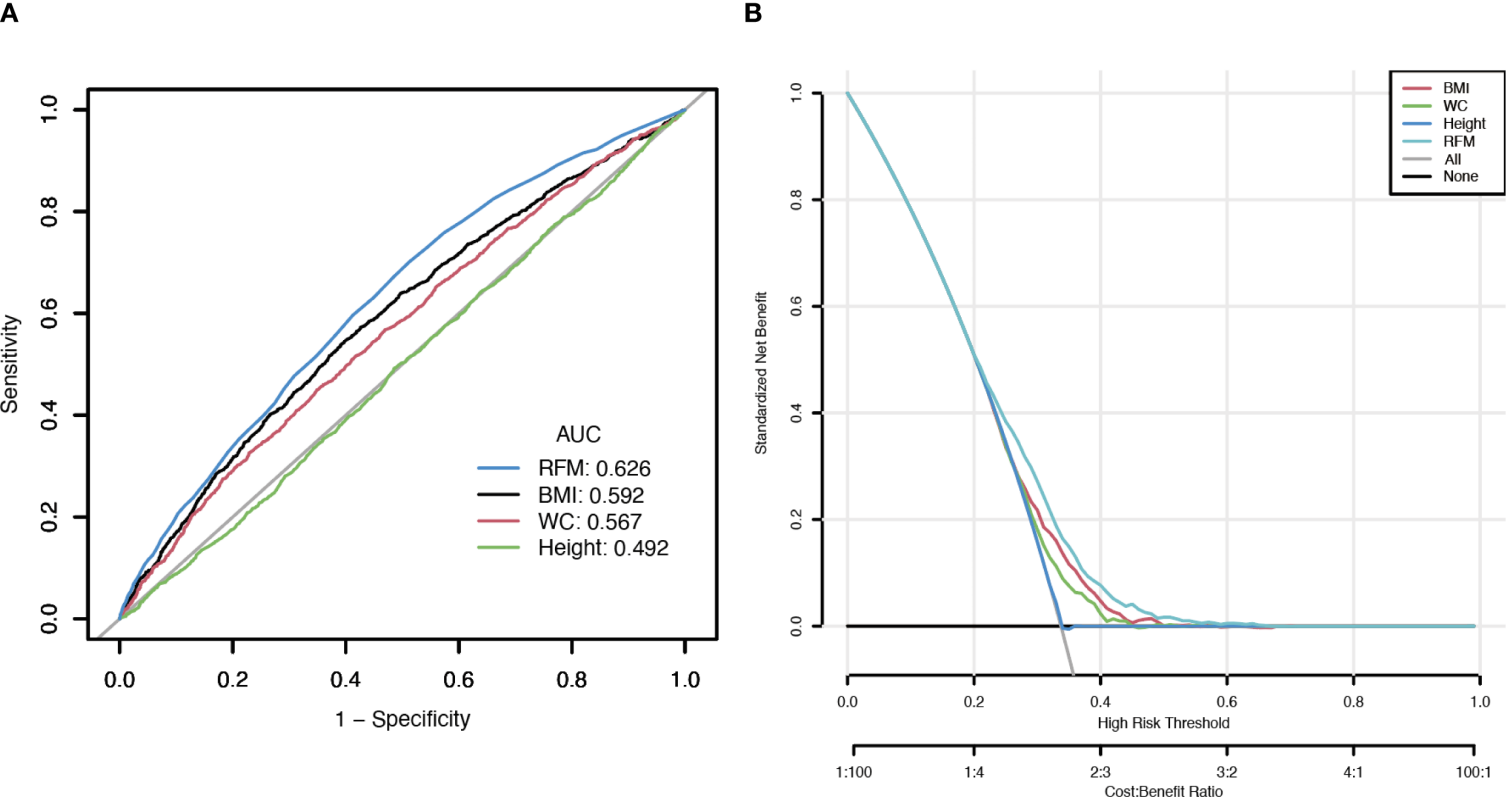
Clinical utility comparison of RFM, BMI, WC, and height. (**A**, The results of ROC analysis; **B**, The results of DCA analysis).

According to the results of the DCA and ROC analysis, RFM showed superior diagnostic and predictive ability for Vitamin D deficiency compared to BMI, WC, and height (**Figures 4A, B**). According to ROC analysis, the area under the curve (AUC) values were 62.6% for RFM, 59.2% for BMI, 56.7% for WC, and 49.2% for height (DeLong’s test: RFM vs. BMI, *P* < 0.001; RFM vs. WC, *P* < 0.001; RFM vs. height, *P* < 0.001) (**Figure 4A**). DCA demonstrated that RFM had a higher net benefit threshold compared to BMI, WC, and height (**Figure 4B**).

### 3.5 Validation of external dataset

In addition, the association between RFM level and the risk of vitamin D deficiency was further evaluated based on adult diabetic patients from the Affiliated Wujin Hospital to Jiangsu University (**Supplementary Material**). Vitamin D deficiency prevalence also increased with rising RFM levels in Chinese diabetic cohort (P<0.001). In Model 1 (no adjusted), Model 2 (adjusting for age, sex, smoking status, and alcohol use), and Model 3 (adjusting for age, sex, smoking status, alcohol use, hypertension, CVDs, HbA1c, TG, LDL-c, HDL-c, and Scr), Logistic regression indicated the ORs and 95% CIs for vitamin D deficiency risk were 1.111 (1.066-1.158), 1.188 (1.089-1.296), and 1.183 (1.080-1.295), respectively (**Supplementary Material**). Additionally, linear regression analysis also discovered a close correlation between RFM and 25(OH)D levels (Model 1: β= -0.261, 95%CI= -0.396–0.126, P<0.001; Model 2: β= -0.510, 95%CI= -0.769- -0.251, P<0.001; Model 3: β= -0.532, 95%CI= -0.809–0.256, P<0.001) (**Supplementary Material**).

## 4 Discussion

This study comprehensively explored the association between RFM and vitamin D deficiency among adults with diabetes, utilizing large-scale national survey data and independent external validation. Our key findings indicate that higher RFM is significantly associated with an increased risk of vitamin D deficiency, and that RFM outperforms traditional anthropometric indicators such as BMI and WC in identifying vitamin D deficiency within diabetic populations.

Accurately deciphering the “obesity paradox” continues to challenge epidemiological studies, primarily due to the complicated interrelations among measures of body fat (5, 26, 27). RFM is simpler to calculate than other mathematically complex obesity indicators, making it more practical for use in broad public health contexts (28). Evidence from earlier studies suggests that RFM is effective in estimating body composition and predicting conditions associated with obesity (10, 14). Especially in patients with type 2 diabetes, characterized by increased visceral and ectopic fat deposits, RFM provides superior sensitivity and specificity in identifying individuals with higher fat mass (29). Since vitamin D is sequestered in fat tissue due to its fat solubility, higher fat mass frequently results in reduced serum vitamin D (30). By more precisely quantifying actual body fat, RFM shows a stronger link to serum 25(OH)D concentration. To enhance interpretability, we applied SHAP, which assigns each predictor a participant-level contribution score indicating whether it raises or lowers risk relative to the average. In our analysis, RFM showed high relative importance and contributed positively, consistent with the higher predicted risk of vitamin D deficiency. Additionally, results from both ROC and DCA analyses support the notion that RFM is a more effective tool than BMI or WC in evaluating vitamin D deficiency. An AUC of 0.626 for RFM denotes modest discrimination, supporting its use to aid risk stratification and testing prioritization for 25(OH)D rather than as a stand−alone screening or diagnostic tool. Notably, our study reveals an inverse relationship between age and the likelihood of vitamin D deficiency, differing from earlier research that identified older adults as more susceptible. Age and vitamin D deficiency might be prone to nonlinear associations. Similar findings have also been reported in studies focusing on the U.S. population (31–34). According to Hongfei Mo et al., younger individuals exhibit a higher deficiency risk due to faster metabolism and increased vitamin D utilization (31). Elevated adiposity levels may intensify vitamin D deficiency through multiple pathways. Firstly, as mentioned above, as a lipophilic compound, vitamin D tends to sequester in adipose tissue, thereby reducing its circulating bioavailability (35, 36). Secondly, higher RFM often reflects lower levels of physical activity and less exposure to sunlight in individuals, both of which are essential for the body’s endogenous production of vitamin D (36). Chronic low-grade inflammation and insulin resistance may act as mediators of the above 2 factors (37–39). On the other hand, Roizen et al. propose that, in obesity, decreased hepatic expression of CYP2R1, the key enzyme for vitamin D 25-hydroxylation, impairs the conversion of vitamin D to 25(OH)D and thus contributes to vitamin D deficiency (40).

Although this study utilized large cross-sectional datasets such as NHANES and incorporated external validation in a Chinese diabetes cohort, indicating a certain degree of representativeness and generalizability, several limitations remain. First, the cross-sectional design precludes any causal inference between RFM and vitamin D deficiency, allowing only an assessment of association. Prospective cohort studies are required to further establish causality. Second, there is no universal consensus on serum 25(OH) D cutoffs. Major organizations-including the Institute of Medicine, the Endocrine Society, the American Geriatrics Society, and others-apply differing thresholds and decision frameworks (21). This lack of standardization complicates cross-study comparisons and may partly account for variability in reported prevalence and effect sizes. Third, although RFM performed better than BMI and WC in our analyses, it is not a gold standard for body composition and does not capture visceral fat or muscle mass. Moreover, we did not fully adjust for key confounders-seasonal variation in vitamin D levels, latitude, vitamin D supplementation, outdoor activity, dietary patterns, and skin exposure-which may have affected the results.

## 5 Conclusion

In conclusion, RFM’s advantage over BMI and WC in assessing vitamin D deficiency risk supports adopting more precise fat indicators, especially in diabetic populations.

## Data Availability

Publicly available datasets were analyzed in this study. This data can be found here: the NHANES database (https://wwwn.cdc.gov/nchs/nhanes) and the figshare database (https://doi.org/10.6084/m9.figshare.29474501.v1).
