# Supplementary material for: Relative fat mass is associated with vitamin D deficiency in individuals with diabetes: evidence from NHANES and a Chinese cohort
Source: Front Endocrinol (Lausanne). 2025 Oct 1;16:1659361. doi: 10.3389/fendo.2025.1659361 (PMC12520909; doi:10.3389/fendo.2025.1659361)
Supplement: Supplementary file 1 [file Table1.docx]

**Table 1. Baseline characteristics of diabetic participants according to vitamin D deficiency.**

| **Characteristic** | **Overall (n=5128)** | **Non-Vitamin D deficiency (n=3398)** | **Vitamin D deficiency (n=1730)** | ***P* value** |
| --- | --- | --- | --- | --- |
| Age (years) | 62.00 (52.00-71.00) | 64.00 (54.00-72.00) | 59.00 (48.00-66.00) | <0.001 |
| Sex, % |  |  |  | 0.051 |
| Female | 2415 (46.87%) | 1572 (45.69%) | 843 (49.88%) |  |
| Male | 2713 (53.13%) | 1826 (54.31%) | 887 (50.12%) |  |
| Race, % |  |  |  | <0.001 |
| Mexican American | 922 (10.00%) | 543 (8.02%) | 379 (15.03%) |  |
| Non-Hispanic Black | 1278 (14.25%) | 642 (9.62%) | 636 (26.03%) |  |
| Non-Hispanic White | 1733 (60.61%) | 1389 (67.77%) | 344 (42.39%) |  |
| Other Hispanic | 593 (6.06%) | 387 (5.34%) | 206 (7.90%) |  |
| Other Race | 602 (9.08%) | 437 (9.24%) | 165 (8.66%) |  |
| Education level, % |  |  |  | 0.007 |
| <High school | 1745 (23.16%) | 1127 (21.76%) | 618 (26.74%) |  |
| High school | 1168 (24.44%) | 772 (24.48%) | 396 (24.32%) |  |
| >High school | 2215 (52.40%) | 1499 (53.76%) | 716 (48.94%) |  |
| PIR | 1.89 (1.09-3.29) | 1.99 (1.12-3.45) | 1.72 (1.01-2.89) | <0.001 |
| Smokers, % | 2550 (50.84%) | 1692 (51.35%) | 858 (49.56%) | 0.423 |
| Alcohol use, % | 3564 (73.79%) | 2377 (75.00%) | 1187 (70.71%) | 0.009 |
| Hypertension, % | 3637 (69.40%) | 2444 (69.89%) | 1193 (68.14%) | 0.314 |
| CVDs, % | 1233 (23.28%) | 843 (23.84%) | 390 (21.86%) | 0.338 |
| BMI (kg/m2) | 31.20 (27.20-36.10) | 30.43 (26.80-35.07) | 32.70 (28.30-38.10) | <0.001 |
| WC (cm) | 107.65 (98.00-119.00) | 106.60 (97.00-117.40) | 110.20 (100.20-122.20) | <0.001 |
| Height (cm) | 166.00 (158.60-173.30) | 166.00 (158.30-173.30) | 165.90 (159.10-173.28) | 0.298 |
| HbA1c (%) | 6.80 (6.30-7.90) | 6.80 (6.20-7.70) | 7.00 (6.40-8.60) | <0.001 |
| TG (mg/dL) | 144.00 (105.00-201.00) | 142.00 (105.00-195.24) | 148.51 (106.58-213.03) | <0.001 |
| TC (mg/dL) | 179.00 (153.00-212.00) | 176.00 (151.00-208.00) | 187.00 (156.00-219.00) | <0.001 |
| LDL-c (mg/dL) | 100.59 (79.00-129.88) | 98.00 (76.62-125.00) | 107.15 (84.00-138.03) | <0.001 |
| HDL-c (mg/dL) | 45.00 (38.00-55.00) | 46.00 (39.00-56.00) | 45.00 (38.00-54.00) | 0.002 |
| SCr (umol/L) | 77.79 (63.65-96.36) | 79.56 (65.42-98.12) | 74.70 (61.22-91.94) | 0.013 |
| RFM | 37.74 (31.88-46.19) | 37.19 (31.60-45.62) | 38.85 (32.52-47.17) | <0.001 |

Continuous data are presented as median and interquartile range, categorical data as unweighted counts and weighted percentages.

Abbreviation: PIR, family income-to-poverty ratio; CVDs, cardiovascular diseases; BMI, body mass index; WC, waist circumference; TG, triglycerides; TC, total cholesterol; LDL-c, low-density lipoprotein cholesterol; HDL-c, high low-density lipoprotein cholesterol; SCr, serum creatinine; RFM, relative fat mass

| **Table 2. Baseline characteristics of diabetic participants stratified by RFM quartiles.** | | | | | |
| --- | --- | --- | --- | --- | --- |
| **Characteristic** | **Q1** | **Q2** | **Q3** | **Q4** | ***P*** value |
| Age (years) | 62.00 (52.00-71.00) | 64.00 (53.00-72.00) | 62.00 (52.00-71.00) | 60.00 (51.00-69.00) | <0.001 |
| Sex, % |  |  |  |  | <0.001 |
| Female | 6 (0.69%) | 78 (5.31%) | 1049 (77.86%) | 1282 (100.00%) |  |
| Male | 1276 (99.31%) | 1204 (94.69%) | 233 (22.14%) | 0 (0.00%) |  |
| Race, % |  |  |  |  | <0.001 |
| Mexican American | 211 (9.95%) | 233 (10.32%) | 213 (8.47%) | 265 (11.15%) |  |
| Non-Hispanic Black | 320 (13.66%) | 280 (10.89%) | 326 (15.70%) | 352 (16.73%) |  |
| Non-Hispanic White | 384 (57.58%) | 518 (66.21%) | 401 (57.89%) | 430 (60.34%) |  |
| Other Hispanic | 125 (5.63%) | 141 (5.63%) | 165 (6.67%) | 162 (6.31%) |  |
| Other Race | 242 (13.18%) | 110 (6.94%) | 177 (11.27%) | 73 (5.47%) |  |
| Education level, % |  |  |  |  | 0.049 |
| <High school | 414 (22.12%) | 427 (20.88%) | 462 (26.39%) | 442 (23.33%) |  |
| High school | 305 (23.05%) | 275 (23.04%) | 284 (24.57%) | 304 (26.92%) |  |
| >High school | 563 (54.83%) | 580 (56.08%) | 536 (49.03%) | 536 (49.75%) |  |
| PIR | 2.04 (1.19-3.60) | 2.08 (1.19-3.64) | 1.74 (1.06-3.05) | 1.67 (0.97-2.78) | <0.001 |
| Smokers, % | 749 (55.88%) | 772 (59.77%) | 494 (43.02%) | 535 (44.86%) | <0.001 |
| Alcohol use, % | 1068 (84.84%) | 1059 (85.60%) | 729 (63.48%) | 708 (61.95%) | <0.001 |
| Hypertension, % | 790 (57.98%) | 952 (73.40%) | 918 (69.36%) | 977 (75.64%) | <0.001 |
| CVDs, % | 268 (19.46%) | 384 (29.16%) | 282 (20.64%) | 299 (23.35%) | 0.001 |
| BMI (kg/m2) | 26.60 (24.36-28.50) | 33.00 (30.40-35.75) | 29.20 (26.20-33.38) | 37.30 (33.89-42.05) | <0.001 |
| WC (cm) | 98.00 (92.43-103.30) | 115.85 (109.40-122.00) | 99.70 (93.03-107.00) | 117.55 (111.00-126.50) | <0.001 |
| Height (cm) | 172.20 (167.20-177.60) | 171.90 (166.40-177.88) | 160.50 (155.00-166.67) | 158.70 (153.80-163.60) | <0.001 |
| HbA1c (%) | 6.80 (6.20-8.00) | 6.80 (6.30-7.80) | 6.77 (6.20-7.90) | 6.90 (6.30-8.00) | 0.320 |
| TG (mg/dL) | 129.00 (93.00-191.00) | 156.10 (113.47-219.80) | 140.59 (104.00-190.95) | 150.67 (114.00-202.68) | 0.002 |
| TC (mg/dL) | 176.00 (149.00-210.00) | 170.00 (146.00-205.00) | 184.00 (159.00-216.75) | 186.72 (161.00-216.00) | <0.001 |
| LDL-c (mg/dL) | 99.10 (76.42-129.42) | 95.00 (73.74-123.47) | 104.00 (82.53-134.20) | 104.68 (83.18-131.00) | <0.001 |
| HDL-c (mg/dL) | 45.00 (38.00-54.00) | 41.00 (35.00-49.00) | 48.50 (41.00-59.00) | 47.00 (40.00-56.00) | <0.001 |
| SCr (umol/L) | 84.86 (72.49-102.54) | 86.63 (73.59-106.08) | 70.72 (57.46-86.63) | 68.31 (57.46-83.10) | <0.001 |
| RFM | 29.12 (27.02-30.61) | 34.33 (33.07-35.82) | 42.85 (40.32-44.78) | 48.99 (47.48-50.67) | <0.001 |
| 25(OH)D (nmol/L) | 63.55 (46.40-82.38) | 60.75 (44.40-77.20) | 63.40 (44.40-84.50) | 58.15 (40.32-80.57) | <0.001 |
| Vitamin D deficiency, % | 380 (23.20%) | 431 (27.55%) | 404 (26.33%) | 515 (35.07%) | <0.001 |

Continuous data are presented as median and interquartile range, categorical data as unweighted counts and weighted percentages.

Abbreviation: PIR, family income-to-poverty ratio; CVDs, cardiovascular diseases; BMI, body mass index; WC, waist circumference; TG, triglycerides; TC, total cholesterol; LDL-c, low-density lipoprotein cholesterol; HDL-c, high low-density lipoprotein cholesterol; SCr, serum creatinine; RFM, relative fat mass

| **Table 3.** Logistic regression models assessing the association between RFM Levels and the risk of vitamin D deficiency. | | | |
| --- | --- | --- | --- |
| Vitamin D deficiency | OR 95%CI | | |
|  | Model 1 | Model 2 | Model 3 |
| Continuous |  |  |  |
| RFM | 1.018 (1.011, 1.026) <0.001 | 1.057 (1.041, 1.073) <0.001 | 1.056 (1.039, 1.073) <0.001 |
| Quantiles |  |  |  |
| Quartile 1 | reference | reference | reference |
| Quartile 2 | 1.202 (1.018, 1.420) 0.030 | 1.394 (1.166, 1.666) <0.001 | 1.385 (1.153, 1.663) <0.001 |
| Quartile 3 | 1.092 (0.923, 1.292) 0.304 | 1.749 (1.323, 2.313) <0.001 | 1.707 (1.280, 2.278) <0.001 |
| Quartile 4 | 1.594 (1.353, 1.878) <0.001 | 2.724 (1.972, 3.763) <0.001 | 2.642 (1.883, 3.708) <0.001 |
| ***P*** for trend | <0.001 | <0.001 | <0.001 |
| OR: odds ratio.  95% CI: 95% confidence interval.  Model 1: non-adjusted.  Model 2: adjusted for age, sex, race, PIR, education, smoking status, and alcohol use.  Model 3: adjusted for age, sex, race, PIR, education, smoking status, alcohol use, hypertension, CVDs, HbA1c, TG, LDL-c, HDL-c, and SCr. | | | |

| **Table 4.** Linear regression analysis of the relationship between RFM and serum 25(OH)D Levels. | | | |
| --- | --- | --- | --- |
| 25(OH)D levels (nmol/L) | β 95%CI | | |
|  | Model 1 | Model 2 | Model 3 |
| Continuous |  |  |  |
| RFM | -0.115 (-0.212, -0.017) 0.022 | -0.708 (-0.888, -0.528) <0.001 | -0.662 (-0.852, -0.471) <0.001 |
| Quantiles |  |  |  |
| Quartile 1 | reference | reference | reference |
| Quartile 2 | -2.489 (-4.770, -0.209) 0.032 | -4.236 (-6.390, -2.081) <0.001 | -3.940 (-6.121, -1.760) <0.001 |
| Quartile 3 | 0.808 (-1.472, 3.089) 0.487 | -7.522 (-10.961, -4.084) <0.001 | -6.925 (-10.419, -3.431) <0.001 |
| Quartile 4 | -3.775 (-6.055, -1.494) 0.001 | -12.679 (-16.640, -8.718) <0.001 | -11.736 (-15.826, -7.646) <0.001 |
| ***P*** for trend | 0.029 | <0.001 | <0.001 |
| 95% CI: 95% confidence interval.  Model 1: non-adjusted.  Model 2: adjusted for age, sex, race, PIR, education, smoking status, and alcohol use.  Model 3: adjusted for age, sex, race, PIR, education, smoking status, alcohol use, hypertension, CVDs, HbA1c, TG, LDL-c, HDL-c, and SCr. | | | |
